# Prophylactic intraoperative tranexamic acid administration and postoperative blood loss after transapical aortic valve implantation

**DOI:** 10.1186/s13019-015-0246-5

**Published:** 2015-03-28

**Authors:** Navid Madershahian, Maximilian Scherner, Roman Pfister, Tanja Rudolph, Antje C Deppe, Ingo Slottosch, Elmar Kuhn, Yeong-Hoon Choi, Thorsten Wahlers

**Affiliations:** 1 Department of Cardiothoracic Surgery, Cologne University Heart Center, Kerpener Strasse 62, D-50937 Cologne, Germany; 2 Department of Internal Medicine III, Cologne University Heart Center, 50937 Cologne, Germany

**Keywords:** Aortic valve stenosis, Tranexamic acid, Blood loss, Blood transfusion

## Abstract

**Objectives:**

Antifibrinolytics are widely used in cardiac surgery to save blood perioperatively. In the present study we evaluated the hemostatic effects of tranexamic acid (TXA) to decrease bleeding tendency and transfusion requirements in high-risk patients following transapical aortic valve implantation (TA-AVI).

**Methods:**

A retrospective analysis was performed on aortic stenosis patients undergoing TA-AVI with or without intraoperative TXA administration to determine postoperative blood loss and transfusion requirements. From January 2009 to August 2010 in total 92 patients were treated without intraoperative TXA administration, from August 2010 to July 2011 54 patients received TXA intraoperatively.

**Results:**

Early postoperative (24 h) blood loss was significantly lower in TXA-group than in non-TXA group (327 ± 274 mL vs. 481.1 ± 318.8 mL; p = 0.003). In the TXA group 53.7% of patients received allogeneic blood products during the hospital stay as compared to 72.8% in the non-TXA group (p = 0.242). TXA group required fewer transfusions (2.1 ± 1.9 vs. 2.9 ± 3.5 Units; p = 0.046) and had no increased incidence of thrombotic or neurological complications. There was no significant difference in the length of ICU, hospital stay, or 30-day mortality. Administration of tranexamic acid was found to be significantly associated with lower blood loss postoperatively (p = 0.002). Furthermore, there was a significant correlation between the postoperative blood loss (p = 0.036) and red blood cell transfusion (p = 0.001) with 30-day mortality.

**Conclusion:**

Low dose prophylactic intraoperative administration of tranexamic acid appears to be effective in reducing postoperative bleeding and the need for allogeneic blood products following TA-AVI.

## Background

At present the majority of cardiac surgical procedures are performed with extracorporeal circulation support. Excessive perioperative bleeding is most common among the well-known complications of cardiopulmonary bypass (CPB) [1]. Off-pump surgery is associated with a reduced frequency of hemorrhagic disorders.

Homologous blood transfusion is commonly used to correct blood loss in surgical patients. However, it is associated with a risk of infection, viral transmission, fluid overload and high costs [2]. Today, anti-fibrinolytic drugs are widely used, particularly in cardiac surgery. Previous reviews have confirmed anti-fibrinolytics to be effective in reducing blood loss and the need for transfusion. Tranexamic acid (TXA) is a synthetic anti-fibrinolytic drug that inhibits the lysine-binding site of plasmin and plasminogen and reduces fibrinolysis and platelet-mediated platelet dysfunction [3-5]. Several studies with TXA have demonstrated a significant reduction of perioperative bleeding and the need for transfusions in on-pump as well as off-pump cardiac surgery [6]. However, the efficacy of TXA in off-pump minimally invasive aortic valve surgery patients has not been investigated yet.

In the present study, we retrospectively evaluated the impact of prophylactic intraoperative tranexamic acid administration on postoperative blood loss and transfusion requirements in high-risk aortic stenosis patients scheduled for transapical aortic valve implantation (TA-AVI).

## Methods

### Patient characteristics

We performed a retrospective chart review of all patients (n = 151) who underwent TA-AVI at our institution between January 2009 to May 2011. Whether a patients was accepted tor TA-AVI was decided by the institutional heart team, considering the patient’s frailty, the preoperative cardiac status, and pre-existing comorbidities and therefore the risk profile of the patients individually.

Patients with the following criteria were excluded: cardiopulmonary bypass support during the procedure or conversion to a conventional aortic valve replacement. This results in a final study population of 146 patients. We analyzed the two groups with regard to the intraoperative use of TXA:

I. TXA-group: TXA during the TA-AVI procedure (n = 54)
II. Non-TXA-group: control group (no TXA during the TA-AVI procedure, n = 92).

The primary outcome variable for this study was the volume of perioperative blood loss and amount of red blood cell (RBC) transfusion. Secondary endpoints included length of intensive care unit stay and total hospital stay and 30-day mortality.

### Prosthetic valve system and procedure

The implanted prosthetic valve system consists of a pericardial xenograft mounted in a balloon expandable stainless steel stent (SAPIEN THV; Edwards Lifesciences, Irvine, CA, USA). All patients received a 23 mm, 26 mm, or 29 mm external diameter prosthesis, respectively, depending on the echocardiographically determined aortic annulus diameter. At the beginning of each procedure heparin was administered (150 IU/kg) and heparin was antagonized with protamine at a 1:1 ratio by the end of surgery. A cell separator was not used. No blood was re-transfused intraoperatively. The chest tube drainages were placed before the chest closure.

### Tranexamic acid administration

From October 2010 the standard protocol at our institution included the low-dose administration of tranexamic acid, 1 g bolus dose before skin incision followed by a continuous infusion of 400 mg/h until the end of the procedure which was proven effective in a large series of patients [7].

### Transfusion criteria

Bleeding and other complications were adjudicated according to the recommendations by the Valve Academic Research Consortium (VARC) [8]. Anemia was defined as a hemoglobin (Hb) level < 13 g/dl in men and < 12 g/dl in women according to the definition by the World Health Organization (WHO). The criteria for the transfusion of packed red blood cells were:

1. bleeding caused hemodynamic instability or
2. hemoglobin concentrations below 8.0 g/dL in the early postoperative period.

In all other situations the decision to transfuse was left to the discretion of the treating physician. The criteria for transfusion of platelet concentrates and fresh frozen plasma were:

1. excessive bleeding and a platelet count < 70.000/μL or
2. a prothrombin time and/or activated partial thromboplastin time of > 1.5 times the upper limit of normal (after heparin reversal), respectively.

Additional protamine was administered in cases of prolonged ACT (the preoperatively measured ACT served as reference).

### Data collection and statistical analysis

The data for all patients’ demographics, clinical characteristics, comorbid conditions, medical treatments, laboratory data, angiographic data, and perioperative and postoperative events were routinely collected in a computerized database for all patients undergoing TA-AVI at our institution prospectively. We used these existing data for our analysis.

Statistical analysis was performed using the StatsDirect (StatsDirect Ltd., Chelsire, UK) and SPSS 20 statistical software packages (IBM, Somers, NY, USA). Continuous variables were compared using the unpaired t-test for parametric variables (in case of Gaussian distribution by visual inspection) or the Mann–Whitney U test for non-parametric (no Gaussian distribution by visual inspection) variables, respectively. A p-value < 0.05 was considered to indicate statistical significance. Tabular data were expressed as mean ± one standard deviation, whereas figures represent mean ± standard error of mean.

The investigators initiated the study, had full access to the data, analyzed the data, and wrote the manuscript. All authors vouch for the data and analysis.

The linear regression analyses of relevant variables were performed in order to identify the predictors for postoperative bleeding, red blood cell transfusion, and 30-day mortality. The regression coefficients (beta) and the corresponding 95% confidence intervals are reported and the p-values < 0.05 were considered to indicate statistical significance. For the endpoints bleeding on postoperative day (POD) 1, and transfusion, the regression coefficients and standard errors are given. For the endpoint mortality, we report the odds ratios and confidence intervals. The limited number of enrolled patients did not allow for extensive multivariate regression analysis.

## Results

### Patients’ characteristics

There were no statistically significant differences between the groups with regard to the demographic and preoperative data (Table 1). The comparisons of intra- and post-procedural characteristics are presented in Table 2. There were no statistically significant differences between groups in terms of the length of the procedure (Non-TXA group 100.0 ± 29.0 min, TXA group 99.2 ± 27.0 min; p = 0.354), the rethoracotomy rate, the renal function parameters, the need for hemodialysis, and major neurologic or thromboembolic complications. The analysis of the postoperative morbidity in terms of length of ICU stay and hospital stay as well as postoperative mortality with regard to death and the 30-day mortality also revealed no significant difference between the two groups (11% in the TXA group vs. 12% in the non-TXA group (p = 0.988)).

**Table 1:** **Comparison between the demographic, clinical and biochemical parameters of the patients with and without intraoperative TXA administration, preoperatively**

	Total	TXA-group	Non-TXA-group	*P*
	(n = 146)	(n = 54)	(n = 92)	
**Female sex (n) %**	(90) 62	(28) 51	(62) 67	*0.07*
**Age (years)**	81.5 ± 6.6	81.4 ± 6.2	81.7 ± 6.8	*0.12*
**BMI (kg/m** ^**2**^ **)**	27.0 ± 4.4	27.2 ± 3.7	26.9 ± 4.4	*0.821*
**Logistic ES (%)**	28.7 ± 18.8	27.4 ± 19.5	29.3 ± 18.6	*0.606*
**STS score (%)**	12.1 ± 5.0	12.1 ± 5.2	12.1 ± 4.9	*0.989*
**Ejection fraction (%)**	54.7 ± 15.7	55.2 ± 17.1	55.5 ± 14.3	*0.402*
**Diabetes mellitus, (n) %**	(49) 34	(15) 28	(45) 49	*0.110*
**CAD (n) %**	(84) 59	(32) 59	(52) 56	*0.540*
**COPD (n) %**	(42) 29	(15) 28	(27) 29	*0.936*
**Previous heart surgery (n) %**	(36) 25	(14) 26	(22) 24	*0.549*
**Aspirin (n) %**	(82) 56	(25) 46	(57) 62	*0.213*
**Pre-op serum Crea (mg/dL)**	1.2 ± 0.5	1.3 ± 0.5	1.1 ± 0.5	*0.074*
**Platelet count (10** ^**3**^ **/mm** ^**3**^ **)**	243.7 ± 43.5	238.4 ± 80.9	250.1 ± 56.6	*0.336*
**Pre-op Hb (g/dL)**	12.3 ± 1.6	12.5 ± 1.6	12.2 ± 1.6	*0.398*
**Pre-op Hct (%)**	37.0 ± 5.1	37.4 ± 4.6	37.1 ± 4.2	*0.778*
**PTT**	27.5 ± 7.4	27.5 ± 4.4	28.0 ± 8.2	*0.296*
**Quick**	96.4 ± 18.6	94.8 ± 20.1	97.6 ± 18.9	*0.304*

BMI: body mass index, CAD: coronary artery disease; COPD,: chronic obstructive pulmonary disease, Hb: hemoglobin, Hct: hematocrit, ICU: intensive care unit; NYHA: New York Heart Association, PTT: partial thromboplastin time, STS: Society of Thoracic Surgeons, TXA, tranexamic acid.

**Table 2:** **Comparison of intra- and post-procedural characteristics**

	Total	TXA-group	Non-TXA-group	*p*
	(n = 146)	(n = 54)	(n = 92)	
**Time of operation (min)**	98.3 ± 27.9	99.2 ± 27.0	100.0 ± 29.0	0.354
**Rethoracotomy (n) %**	(11) 7	(2) 4	(9) 10	0.221
**Serum Crea 72 h after TA-AVI (mg/dL)**	1.5 ± 0.8	1.4 ± 0.8	1.4 ± 0.8	0.436
**Need for haemodialysis (n) %**	(15) 10	(5) 9	(10) 11	0.751
**Major neurologic deficit (n) %**	(3)	(1) 2	(2) 2	0.946
Major thrombembolic complication (n) %*	(2) 1	(1) 2	(1) 1	0.662
**ICU stay (day)**	4.2 ± 3.0	4.3 ± 3.2	4.2 ± 3.0	0.909
**Hospital stay (day)**	11.8 ± 5.8	10.8 ± 4.4	11.9 ± 5.6	0.586
**30-day mortality (n) %**	(17) 11	(6) 11	(11) 12	0.988

Hb: Hemoglobin; ICU: intensive care unit; TA-AVI: transapical aortic valve implantation.

*One episode of pulmonary embolism occurred in either group on 4^th^ and 5^th^ POD.

### Bleeding complications

The postoperative RBC transfusion requirements are summarized in Table 3. The mean postoperative blood loss in the TXA group was significantly reduced compared to the Non-TXA group 24 hours after TA-AVI (327.0 ± 274.5 mL vs. 481.1 ± 318.8 mL; p = 0.003). However, there was no statistical significance in terms of average blood loss during the following 24 hours (175 ± 152 mL vs. 219.1 ± 197.9 mL; p = 0.727). Regarding blood transfusions during the peri-procedural period, the patients without TXA had blood transfusions more frequently (2.1 ± 1.9 units vs. 2.9 ± 3.5 units; p = 0.046). Only 24.1% of tranexamic acid patients (n = 13) received allogeneic blood products during the hospital stay versus 45% of control patients (n = 41, p = 0.013).

**Table 3:** **Postoperative blood transfusion requirements**

	Total	TXA-group	Non-TXA-group	*P*
	(n = 146)	(n = 54)	(n = 92)	
**Blood loss 24 h after TA-AVI (mL)**	425 ± 310	327 ± 274	481.1 ± 318.8	0.003
**Blood loss 48 h after TA-AVI (mL)**	214 ± 217	175 ± 152	219.1 ± 197.9	0.727
**Number of RBC transfused patients (n/%)**	54/68.7	13/24.1	41/44.6	0.013
**Total units in hospital (units)**	2.5 ± 3.1	2.1 ± 1.9	2.9 ± 3.5	0.046

### Predictors of bleeding (Univariate Analysis)

The regression analysis (Table 4) revealed a negative correlation between postoperative blood loss on the POD 1 and the administration of tranexamic acid (p = 0.002). Furthermore, tranexamic acid (p = 0.026) and the preoperative hemoglobin plasma concentration (Hb) (p = 0.009) had an independent negative impact on the frequency of red blood cell transfusion (Table 5). There was no correlation between the postoperative blood loss and duration of operation, preoperative intake of acetylsalicylic acid, or the preoperative INR value (Table 4). According to the 30-day mortality (Table 6) the analysis revealed a significant correlation with red blood cell pack (RBC) transfusion (p = 0.001), postoperative blood loss (p = 0.036), re-thoracotomy rate (p = 0.002), and the logistic EuroSCORE (p = 0.033).

**Table 4:** **Univariate regression analysis for blood loss on the first postoperative day**

	Beta	Standard error	*p*
**Tranexam acid**	- 153.990	50.009	0.002
**OR duration**	0.545	0.908	0.550
**ASS preoperative**	58.656	50.872	0.251
**INR preoperative**	1.199	0.985 - 1.269	0.601

**Table 5:** **Factors influencing the red blood cell transfusion following TA-AVI**

	Beta	Standard error	*p*
**Tranexam acid**	- 1.161	0.518	0.026
**ASS preoperative**	- 0.474	0.521	0.365
**Hb preoperative**	- 0.432	0.164	0.009

**Table 6:** **Risk factors for 30-day mortality following TA-AVI**

	Odds ratio	95% Confidence interval	*p*
**EuroSCORE log**	1.028	1.002 – 1.053	0.033
**STS Score (ROM)**	1.029	0.987 – 1.073	0.176
**EF preoperatively**	1.023	0.988 – 1.058	0.196
**female gender**	2.266	0.700 – 7.334	0.172
**tranexam acid**	0.920	0.320 – 2.648	0.878
**Blood loss (POD 1) (ml)**	1.002	1.000 – 1.003	0.036
**Red blood cell transfusion**	1.360	1.161 – 1.594	<0.001
**rethoracotomy**	8.542	2.267 – 32.187	0.002

## Discussion

The bleeding complications and RBC transfusions remain an issue after transcatheter aortic valve implantation, but their real impact on the patients’ morbidity and mmortality is unknown. Van Mieghem *et al.* [9] sought to evaluate the incidence, the predictors and the clinical impact of bleeding and RBC transfusion in a large retrospective multicenter trial. In this large cohort the authors confirmed RBC transfusion post TAVI to be associated with an increased all cause and cardiac mortality at 30 days and 1 year, including an increased risk for a major stroke and acute renal failure. Furthermore, there is still an important risk related to the transfused blood products, such as the transmission of viral infections, the induction of immunological transfusion reactions, and the suppression of the immune system.

It is well established that tranexamic acid reduces both the postoperative blood loss and the transfusion requirements following on-pump as well as off-pump cardiac surgery [6]. However, to date there is no information about the effects of TXA on transapical aortic valve implantation.

The main results of this study are:

1. Tranexamic acid administered before and during the operation decreases the postoperative blood loss and the need for RBC transfusion
2. The postoperative blood loss and RBC transfusion are associated with an increased 30-day mortality.

Bleeding after TAVI is a frequent complication, occurring in over 25% of patients [10]. Various studies analyzing the predictors of early and late mortality after TAVI found bleeding to be associated with an increased risk for a fatal course. After 2 years of follow-up in the randomized PARTNER trial, major bleeding was associated with a 2-fold risk of mortality [11]. Halliday *et al.* [12] investigated the impact of bleeding on outcome and reported a higher in-hospital and 6-month mortality in patients with life threatening bleeding (LTB) complications. Another multicenter study found LTB to be a predictor of 30-day mortality by multivariate analysis [9]. Tamburino *et al.* [13] compared the 1-year outcomes of patients undergoing TAVI or surgical aortic valve replacement and found LTB to be a strong independent predictor of all-cause mortality at 1 year. At the 3-year follow-up in the CoreValve Italian Registry, LTB was associated with a significantly higher mortality, already being observed at 30 days [14,15]. Our study results are confirm these findings, showing a negative impact of increased postoperative bleeding on 30-day and 1-year survival after TAVI. Bleeding after TAVI was mostly caused by access complications that can lead to hemodynamic instability, increasing the risk of acute kidney injury (AKI) and myocardial ischemia. Generally, bleeding is associated with transfusions, which have deleterious effects by paradox impairment in oxygen delivery and transfusion-related immunomodulation associated with a higher risk of infection [9]. They also increase the incidence of AKI, which rises mortality [16]. The transfusions, especially ≥ 4 RBC units, have an adverse impact on outcome at 1 year [11]. This could be an effect of the transfusions themselves or the transfusions could be a marker of a higher risk status, like baseline anemia. Anemia before TAVI was an independent predictor of 1-year mortality in a cohort of 118 consecutive patients [7]. In our analysis, transfusions and baseline anemia had a negative impact on 1-year mortality. Anemic patients were not more likely to bleed, but they had a higher rate of transfusion. Similar declines in Hb lead to lower final Hb levels in anemic patients, which, in turn, trigger transfusion.

Tranexamic acid has been shown to be effective in reducing blood loss in surgery, particularly cardiac surgery [17,18]. Due to its hemostatic properties and miniscule costs, tranexamic acid is routinely administered in all patients undergoing cardiac surgery with and without CPB. However, there is no clear consensus regarding the optimal dosage of tranexamic acid (dosages between 1 g and > 10 g or 10–100 mg/kg described in the post-aprotinin era [14,19]). Recently, a dose–response relationship between tranexamic acid (TA) dose and seizure has been observed in cardiac surgery patients [16,20,21]. Increased incidence of postoperative generalized seizures have been shown to be associated with high-dose administration of TXA (≥100 mg/kg) [16,20,21].Our low-dose administration of tranexamic acid (1 g as a bolus dose before skin incision followed by a continuous infusion of 400 mg/h until the end of surgery) is much lower compared to other described dosing regimens, but it has been proven effective in a large series of patient [7].

### Limitations

There are certain limitations to this study. The results of this study were obtained from a database with prospectively collected data. However, this is a post hoc non-pre-specified analysis and we cannot rule out the possibility that other potential confounding variables not included in the study might have affected the results. Furthermore, our findings are based on a very small sample size (especially with regard to the possibilities of statistical analysis) and clinical events from a single site. However, this is a homogeneous, well comparable patient group.

One main shortcoming of the present study is that we did not have data regarding the peri-procedural D-dimer and fibrinogen plasma concentrations. Therefore, these shortcomings -due to the retrospective study design with the chance that other potential risk factors have not been included- limit the conclusions that can be drawn. Therefore, a larger prospective randomized trial is needed to overcome these shortcomings.

## Conclusions

In summary, tranexamic acid administered before and during the operation was effective in decreasing both bleeding and transfusions following TA-AVI. However, no direct correlation can be shown between intraoperative administration of tranexamic acid and 30-day mortality. Therefore, further studies with larger series of patients are neededx to confirm these initial data.

## Abbreviations

ACT: Activated clotting time
BMI: Body mass index
CAD: Coronary artery disease
COPD: Chronic obstructive pulmonary disease
CPB: Cardiopulmonary bypass
Hb: Hemoglobin
Hct: Hematocrit
ICU: Intensive care unite
IU: International Units
LTB: Life threatening bleeding
NYHA: New York Heart Association
RBC: Red blood cells
TAVI: Transcatheter aortic valve implantation
TA-AVI: Transapical aortic valve implantation
TXA: Tranexamic acid
